# Updates on the Biological Heterogeneity of Mantle Cell Lymphoma

**DOI:** 10.3390/cancers17040696

**Published:** 2025-02-19

**Authors:** Andrew Ip, Maciej Kabat, Lindsay Fogel, Hassan Alkhatatneh, Jason Voss, Amolika Gupta, Alexandra Della Pia, Lori A. Leslie, Tatyana Feldman, Maher Albitar, Andre H. Goy

**Affiliations:** 1 Department of Oncology, Hackensack Meridian School of Medicine, Nutley, NJ 07110, USA; lindsay.fogel@hmhn.org (L.F.); lori.leslie@hmhn.org (L.A.L.); tatyana.feldman@hmhn.org (T.F.); goy.andre@hmhn.org (A.H.G.); 2 John Theurer Cancer Center, Hackensack Meridian Health, Hackensack, NJ 07601, USA; jason.voss@hmhn.org (J.V.); alexandra.dellapia@hmhn.org (A.D.P.); 3 Department of Internal Medicine, Hackensack University Medical Center, Hackensack, NJ 07601, USA; maciej.kabat@hmhn.org; 4 Hackensack Meridian School of Medicine, Nutley, NJ 07110, USA; 5 Jefferson Einstein Philadelphia Hospital, Philadelphia, PA 19141, USA; hassan.alkhatatneh@jefferson.edu; 6 Inova Fairfax Hospital, Falls Church, VA 22042, USA; 7 Genomic Testing Cooperative, Irvine, CA 92618, USA; maher.albitar@hmhn.org

**Keywords:** mantle cell lymphoma, heterogeneity, risk profiling, NGS, novel therapies, Bruton’s tyrosine kinase inhibitors, relapsed/refractory MCL

## Abstract

Advancements in mantle cell lymphoma (MCL) have illuminated the disease’s molecular diversity, leading to a wide variation in the outcomes observed in MCL. Current prognostic risk scores are continuously revised to incorporate new updates in the mechanistic or biologic understanding of MCL. Nevertheless, key high-risk features of MCL associated with rapid disease progression and poor survival, such as *TP53* mutations, complex karyotypes, and blastoid or pleomorphic morphologies, remain absent from available prognostic tools. The greater accessibility of genomic technologies, such as next-generation sequencing (NGS), has enabled clinicians to identify specific genetic alterations that serve as prognostic signals and disease monitoring parameters, cultivating accurate risk profiling that is illustrative of MCL heterogeneity. Through an increased understanding of distinct MCL behaviors, novel therapies that mechanistically target disease biology, including Bruton’s tyrosine kinase inhibitors, BCL-2 inhibitors, ROR1 inhibitors, and bispecific T-cell engagers, have broadened the treatment armamentarium for relapsed/refractory MCL cases. These interventions, in addition to chemoimmunotherapy and autologous stem cell transplantation mainstays, confer the individualization of treatment and improved survival outcomes. Further exploration of the considerable biological heterogeneity of MCL can enhance knowledge, management, and the treatment of this rare lymphoma subtype.

## Simple Summary

This review article provides an update on the current knowledge surrounding mantle cell lymphoma (MCL), focusing on the disease’s complexity and heterogeneity, as well as its prognostic factors. Advancements in risk stratification scoring systems are reviewed, and the emerging role of genomic technologies and their potential to inform risk profiling are also highlighted. In addition, the article discusses novel therapies, including Bruton’s tyrosine kinase inhibitors, BCL-2 inhibitors, ROR1 inhibitors, and bispecific T-cell engagers, which could become cornerstones of MCL treatment in the future.

## 1. Introduction

Mantle cell lymphoma (MCL) is a rare and notably heterogeneous subtype of non-Hodgkin lymphoma (NHL), accounting for an estimated 3% to 10% of NHL cases, with the median age of diagnosis being 68 years [1,2]. The pathogenesis of MCL is frequently attributed to the chromosomal translocation t(11;14), fusing the *CCND1* gene with the immunoglobulin heavy chain gene, leading to the overexpression of cyclin D1, a crucial regulator of the cell cycle. MCL cells typically express BCL-2 and CD5, but not CD23.

This lymphoma subtype is noted for its variable clinical behaviors, from relatively indolent to highly aggressive forms. Despite advances in treatment, MCL is still generally considered incurable. Most patients present with advanced-stage disease, and with a median overall survival of 3 to 5 years [2,3,4]. However, median overall survival can vary substantially according to patient risk level (2.2 vs. 13.2 years in high- and low-risk patients, respectively) [5].

Historically, the Mantle Cell Lymphoma International Prognostic Index (MIPI), supplemented by the Ki-67 proliferation index, has served as the cornerstone for patient risk stratification. However, the utility of these conventional metrics is somewhat limited, failing to capture the full scope of MCL’s biological diversity. Notably, high-risk features, such as *TP53* mutations, complex karyotype, and blastoid or pleomorphic morphology are not included in the MIPI, yet these features invariably foreshadow a dismal prognosis post-standard chemoimmunotherapy regimens [6,7,8,9]. Amidst the evolving therapeutic paradigm for MCL, underscored by the approval of novel agents, there emerges a need to uncover the biological and molecular idiosyncrasies of MCL. Such an understanding is pivotal for discerning the heterogeneity in treatment responses and survival outcomes. In this article, we review the available literature on the biological heterogeneity of MCL as well as the evolution of risk stratification and management strategies.

## 2. Biologic Heterogeneity and Subclassification of MCL

MCL is commonly subdivided based on its clinical presentation into two types: nodal and leukemic non-nodal MCL. Nodal MCL, which accounts for more than 75% of cases, presents with lymphadenopathy and may also involve the spleen and typically extranodal sites such as the gastrointestinal tract (>90% cases). The extent of lymphadenopathy, whether localized or diffuse, often correlates with the disease’s aggressiveness. In contrast, leukemic non-nodular MCL is generally associated with a more favorable prognosis, and patients typically present with a leukemic phase with no or very minimal lymphadenopathy or splenomegaly. However, these cases can exhibit aggressive behavior, especially after the acquisition of *TP53* mutations over time [9,10,11,12].

The latest revisions of the World Health Organization Classification of Hematolymphoid Tumors (5th edition, 2023 update) and the International Consensus Classification of Mature Lymphoid Neoplasms (2022) have further refined MCL subclassification into three distinct categories: in situ mantle cell neoplasia, classic mantle cell lymphoma, and non-nodular MCL, enhancing both diagnostic and prognostic frameworks (Table 1) [10,11].

In situ mantle cell neoplasia (ISMCN) is characterized by the presence of cyclin D1-positive cells within the mantle zones of lymphoid follicles. Unlike the more common and aggressive forms of MCL, ISMCN is often localized and detected incidentally. Representing an early phase of MCL, many ISMCN cases remain stable and asymptomatic over time, requiring only observation without treatment [9,13,14,15].

The classical subtype of mantle cell lymphoma is associated with progressively worsening lymphadenopathy and an aggressive presentation. Over 95% of cases have the *IGH:CCND1* translocation, which is synonymous with cyclin D1-positive MCL [16,17]. However, there are also less common phenotypes of MCL. The rarer cyclin D1-negative variants use alternative pathways for disrupting cell cycle control, including *CCND2* [18]. High-risk subtypes, such as blastoid and polymorphic variants, often display more aggressive behavior, including a heightened mitotic rate and complex karyotype [19,20,21]. These molecular aberrations contribute to a heightened risk profile, underscoring the importance of tailored therapeutic strategies to address the distinct pathogenic mechanisms and resistance pathways in these subtypes (Table 2).

**Figure 1:**
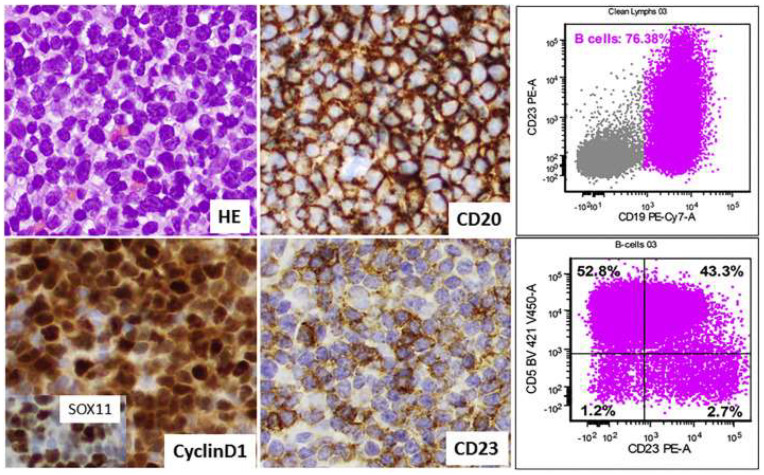
Example of H&E staining, immunohistochemistry, and flow cytometric analysis for a case of leukemic nnMCL by Saksena et al., revealing diffuse growth patterns and CD5, CD19, CD20, CD23, cyclin D1, and SOX11 positivity [23].

Non-nodal MCL (nnMCL) manifests predominantly through extranodal involvement, typically involving the blood, bone marrow, and spleen, with minimal to no lymphadenopathy. Representing 10% to 20% of cases, nnMCL is associated with a more indolent disease course and is characterized by initial presentations of lymphocytosis and splenomegaly [22]. Unlike classical MCL, nnMCL lacks SOX11 expression and exhibits low Ki-67 expression, indicating less aggressive growth (Figure 1). Additionally, it displays a unique genetic profile characterized by fewer genomic alterations and a predilection for *IGV* gene mutations, which is associated with higher rates of somatic DNA hypermutation. These genetic features suggest that nnMCL originates from memory B-cells that have undergone germinal center reactions [2].

This refined subclassification of MCL into ISMCN, classical MCL, and nnMCL facilitates a deeper understanding of MCL’s biological diversity, which is crucial for initial diagnosis, prognosis, therapeutic decision-making, and the development of targeted treatment strategies. Advanced genomic profiling techniques such as next-generation sequencing are poised to further subclassify MCL, enabling clinicians to more accurately predict behavior and treatment responses.

## 3. Traditional Prognostic Markers and Risk Stratification

The prognostic landscape of MCL is shaped by many factors, including molecular subtypes, disease stages, and pathologic features, which highlights the need for precise prognostic markers to guide therapeutic strategies.

The variety of prognostic tools utilized in MCL are outlined in Table 3. In 2008, the MIPI was introduced and has since become pivotal in MCL prognostication. This index was developed to overcome the limitations of the older International Prognostic Index (IPI), which was established in the 1990s for the prognosis of non-Hodgkin’s lymphoma, and to offer a more tailored prognostic framework for MCL [24,25].

Despite the utility of the original MIPI, its capacity to adequately differentiate between low- and intermediate-risk groups was limited, prompting the development of the simplified MIPI (s-MIPI) and biological MIPI (MIPI-b) [24,27]. The s-MIPI was designed for ease of use in the clinical setting, assigning points for different risk factors to streamline patient stratification. Meanwhile, the MIPI-b, which was introduced in the mid-2010s, enhanced the original MIPI model by integrating the Ki-67 proliferation index, a biomarker reflective of tumor proliferation. This inclusion was based on the findings that the proliferation rate of malignant cells, as indicated by Ki-67 staining, provides insight into the tumor’s aggressiveness, with high Ki-67 indices (>30%) being associated with poor outcomes [24,25,27,30]. Yet, the MIPI-b has faced criticism for its inaccurate prognostication in cases with intermediate levels of Ki-67 expression [24,30,31]. In response, new scoring systems were introduced, including the MIPI-B-miR, which incorporates microRNA (miRNA) expression profiles to enhance the traditional MIPI-b tool, and the combined MIPI (MIPI-c) [28,29].

Specifically, the overexpression of miR-18b has been identified as a significant poor prognostic marker in MCL, leading to the development of the MIPI-B-miR model [5]. This prognostic model has had significantly better stratification of patients in all risk groups compared to the standard MIPI-b with respect to overall survival (OS) and progression-free survival (PFS). The MIPI-c, developed in the late 2010s, introduced a binary classification for Ki-67, simplifying the tumor proliferation rate assessment. While this approach reduced the granularity of MIPI-b’s continuous values, it allowed for clearer and more direct clinical decision-making, categorizing patients into distinct risk groups, as follows, that enabled superior discriminative power: low, low-intermediate, high-intermediate, and high [5,29]. The evolution of MIPI to its variants highlights the importance of adapting prognostic instruments to account for emerging research into MCL’s heterogeneity.

Several other high-risk features are not covered by the MIPI and its variants, such as blastoid/pleomorphic morphology, complex karyotype, and specific genetic aberrations (i.e., *TP53*, *NOTCH1*, *KMT2D*, SOX11) [8,32]. Newer scoring systems that harness advancements in genomic technologies have been proposed, such as MIPI-Genetic and MIPI-c with TP53 and/or MCL35, which aim to better identify patients with high-risk disease not captured by the traditional scoring systems [5,33,34,35]. Additionally, emerging research highlights the significance of other biomarkers such as beta-2 microglobulin, which is often elevated in cases with a higher tumor burden. Increased beta-2 microglobulin is associated with a poor prognosis [36], mutations in Bruton’s tyrosine kinase (*BTK*) gene, which can confer resistance to BTK inhibitors [37], and BCL-2 overexpression, which can impact the efficacy of therapies that target apoptotic pathways [38].

Incorporating these biomarkers and others, which are reviewed below, into existing prognostic models, as well as harnessing novel genomic technologies, could enhance the precision of risk stratification, ultimately guiding more personalized treatment approaches in this complex and challenging lymphoma subtype.

### 3.1. Blastoid and Pleomorphic Variants

**Figure 2:**
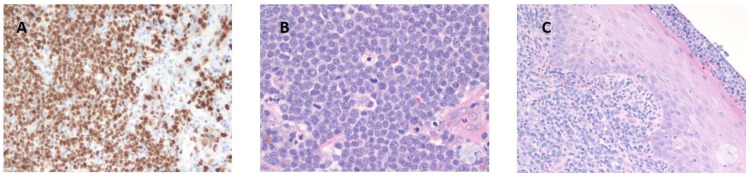
Ki-67 and hematoxylin and eosin staining in a 63-year-old male patient with a blastoid variant of MCL, presenting in the tonsils [42]. (**A**) Ki-67 staining showing a high proliferation index (>90%). (**B**) H&E staining showing medium-sized blastoid cells. (**C**) H&E staining showing medium-sized blastoid cells (higher magnification).

Blastoid (Figure 2) and pleomorphic variants of MCL are aggressive subtypes characterized by rapid disease progression compared to classical MCL. Recent studies indicate that these variants comprise about 10% to 20% of all MCL cases [29,39,40]. A study analyzing outcomes among patients with these variants reported a median OS of 68 months and a median PFS of 38 months [41]. Despite the strong associations between cell morphology and prognosis, this relationship is likely driven by underlying molecular and genetic aberrations, including an elevated Ki-67 index. This elevated proliferation index is frequently observed in blastoid variants and correlates with a more aggressive disease course and poorer prognosis [29].

### 3.2. Complex Karyotype

The presence of a complex karyotype indicates a high degree of genomic instability, a common hallmark of cancer. A complex karyotype is defined as the presence of three or more chromosomal abnormalities within a tumor’s cells and is associated with a significantly shorter OS and PFS compared to patients with a less complex or normal karyotype. This likely results from the cumulative effects of multiple genetic abnormalities, which disrupt key oncogenes and tumor suppressor genes critical for cell cycle regulation [43].

### 3.3. TP53 Abnormalities

The *TP53* gene codes for the tumor suppressor protein p53, which is vital in controlling cell division and cell death. *TP53* aberrations, caused by the deletion of the p arm of chromosome 17 containing the *p53* gene, mutations in the *TP53* gene, or high p53 expression levels as assessed by immunohistochemistry, serve as poor prognostic markers. The European Mantle Cell Lymphoma Network defined low p53 expression as 1–10%, intermediate expression as 10–50%, and high expression as >50%. After adjusting for the MIPI score and Ki-67 index, high *TP53* expression was shown to be a strong prognostic marker for both an inferior time to treatment failure and OS compared with low *TP53* expression. The immunohistochemical overexpression of p53 often correlates with underlying *TP53* mutations because the mutated p53 protein accumulates within cells instead of being degraded [44]. Patients with the disease harboring a *TP53* mutation have a short median OS of 1.8 years [45]. In a multivariate analysis, *TP53* was the only significant independent molecular marker that improved the prognostic value of MIPI [32].

In the European MCL Younger trial, the presence of *TP53* alterations markedly influenced prognosis. Both *TP53* mutations through NGS and deletions of chromosome 17p [del(17p)], which encompasses the *TP53* gene, were initially linked to poorer survival in a univariate analysis. However, after adjusting for additional risk factors, including MIPI, Ki-67 index, *CDKN2A* deletion, blastoid morphology, and more, only *TP53* mutations emerged as an independent predictor for an adverse OS. This distinction highlights that while *TP53* deletions may signal a more aggressive disease overall, it is the mutation that confers the increased risk, underscoring the importance of incorporating *TP53* mutation status into risk assessment and treatment planning [19].

### 3.4. CDKN2A Deletions

*CDKN2A* encodes the tumor suppressor CDKI p16, which inhibits cyclin D1-dependent kinase CDK4 from interacting with cyclin D1 to phosphorylate retinoblastoma tumor suppressor protein (Rb). Mutations in *CDKN2A*, therefore, lead to hyperphosphorylated Rb with subsequent progression through the cell cycle. In newly diagnosed MCL patients with bone marrow involvement of ≥5%, concurrent *TP53* aberrations (i.e., mutation or deletion) and *CDKN2A* deletions conferred a shortened event-free survival of 3 months and an overall survival of 10 months following treatment with frontline immunochemotherapy [46].

### 3.5. SOX11

SOX11 is a transcription factor that is predominantly expressed in the developing central nervous system of embryos and various epithelial tissues. It has been implicated in a range of malignancies, including solid tumors such as breast and ovarian cancers, along with hematologic malignancies like MCL and acute myeloid leukemia. It is expressed in over 90% of cyclin D1-positive MCL cases and acts as a diagnostic marker for cyclin D1-negative MCL [32,47,48]. The prognostic role of SOX11 expression in MCL appears to be limited. Although some studies have suggested that the absence of SOX11 is associated with worse outcomes, its prognostic impact is no longer significant after accounting for factors such as age, ECOG performance, LDH levels, Ki-67 index, and p53 expression [32,47,48].

### 3.6. Cyclin D1 (CCND1)

Cell cycle regulation-related markers such as cyclin D1 are frequently upregulated in MCL. Chromosomal t(11;14) (q13;q32) translocation leads to cyclin D1 mRNA overexpression in pre-germinal center B-cells, which promotes tumor proliferation by dysregulating the cell cycle at the G1-S phase transition. Cyclin D1 not only stimulates tumor growth but also helps cancer cells to evade apoptosis and weaken immune control through its interaction with several other molecular signaling pathways, including B-cell receptor (BCR) and phosphoinositide 3-kinase (PI3K) [49]. Both cyclin D1-positive and negative MCLs generally exhibit similar clinical courses [50,51].

### 3.7. Immunoglobulin Heavy Chain Variable (IGHV) Mutations

MCL is characterized by the presence of the t(11;14) translocation, which juxtaposes the *CCND1* gene with the IGH gene, leading to the overexpression of cyclin D1. Normally, *IGHV* genes undergo somatic hypermutation during B-cell development, a critical factor in antibody diversity [52]. While *IGHV* mutation is classically implicated as a prognostic factor for chronic lymphocytic leukemia (CLL), recent studies have investigated its significance in MCL prognosis. Mutated *IGHV* is typically associated with nnMCL. Patients whose MCL is characterized by significant *IGHV* mutations have been found to exhibit lower chemotherapy response rates and a worse OS [53]. Similarly, *IGHV* mutation load was found to be inversely related to OS [54]. Therefore, *IGHV* mutation status should be considered alongside other factors to inform prognosis and treatment strategies.

### 3.8. ATM

The ataxia telangiectasia mutated gene is a crucial tumor suppressor gene from the PI3K family that encodes a serine–threonine kinase. *ATM* is activated in response to double-strand DNA breaks and induces cell cycle arrest. If the *ATM* gene is mutated, cell cycle checkpoints are overridden, ultimately, leading to chromosomal imbalances and an increased likelihood of oncogenic transformations [49].

An analysis of the whole-exome sequencing of MCL lines to evaluate recurrent MCL mutations revealed that *ATM* mutations were only present in SOX11-positive tumors, hinting that *ATM* alterations are linked to a specific MCL subtype with more aggressive clinical behavior [55]. A recent study evaluated the prognostic role of *ATM* among patients with wild-type *TP53* compared to *TP53* aberrancy. The presence of an *ATM* mutation in wild-type *TP53* patients was associated with a reduced median PFS (38 months vs. 138 months) and a reduced median OS (138 months to 104 months). Among patients with aberrant *TP53*, there was not a statistically significant association between mutated *ATM* and either PFS or OS [56].

### 3.9. NOTCH1 and NOTCH2

*NOTCH1* and *NOTCH2* encode transmembrane receptors that play a critical role in B-cell development and differentiation and also regulate the expression of several proto-oncogenes [57]. Mutations in *NOTCH1* and *NOTCH2* genes have been identified in several B-cell malignancies, as well as approximately 5–10% of MCL cases [57,58]. These mutations result in cellular resistance to degradation, leading to the upregulation of genes responsible for angiogenesis and cell cycle progression [59,60].

*NOTCH1* and *NOTCH2* gene mutations have been associated with more aggressive phenotypes of MCL, such as blastoid and pleomorphic histologies [48], and with shorter survival rates. *NOTCH1* mutation showed a significant decrease in OS (median OS of 1.43 years in *NOTCH1*-mutated patients vs. 3.85 years in *NOTCH1* wild-type patients) [57,61]. *NOTCH2* mutations were associated with a worse MCL prognosis (3-year OS: 0 vs. 62%) [55].

### 3.10. MYC

*MYC* is an oncogene involved in the regulation of growth-promoting signal transduction pathways and various downstream signaling pathways [62]. *MYC* translocations have been identified in many aggressive B-cell lymphomas (e.g., Burkitt lymphomas and double-hit lymphomas). In MCL, *MYC* translocations are less common compared to other B-cell lymphomas but have been associated with blastoid variant morphologies and poor outcomes [63,64,65]. High MYC protein expression was found to be an independent risk factor for worse outcomes, with a median OS of 2.2 years and PFS of 1.8 years, when compared to patients with low MYC expression with a median OS of 7.3 years and PFS of 5.2 years. Patients with high MYC expressing tumors also had a higher risk of death and were noted to have additional risk factors such as an older age at diagnosis, a high Ki-67 proliferation index, a high MIPI score, and *TP53* aberrations [66].

## 4. Newer Genomic Expression Profiling in MCL

NGS has been a revolutionary force in cancer screening and management, providing comprehensive genomic insights into tumors and genetic factors. Its widespread use across various cancer types enables the detection of somatic mutations, inherited cancer-associated germline mutations, and genetic modifiers that influence cancer risk and progression. Recent studies have demonstrated the genetic mutations that are most prevalent in patients with MCL. Notably, a comprehensive systematic review and meta-analysis conducted by Hill et al. assessed 32 genes and their role in the clinical course of MCL. The study found the *ATM* gene exhibited the highest mutation rate at baseline disease (43.5%), followed by *TP53* (26.8%), *CDKN2A* (23.9%), and *CCND1* (20.2%). Upon disease relapse/progression, these rates increased to 57.6% for *ATM*, 43.0% for *TP53*, 29.5% for *CDKN2A*, and 27.7% for *CCND1* [67].

NGS has also proven to be a powerful tool for detecting circulating tumor DNA (ctDNA) in the peripheral blood of patients with diffuse large B-cell lymphoma (DLBCL), serving as an independent prognosis marker. Lahkotia et al.’s study explored ctDNA encoding the immunoglobulin receptor sequences in serum as a prognostic biomarker in MCL [68]. In this prospective phase 2 study, previously untreated MCL patients underwent induction therapy with bortezomib and dose-adjusted etoposide, doxorubicin, and cyclophosphamide with prednisone, vincristine, and rituximab (DA-EPOCH-R), followed by the random assignment to observation or bortezomib maintenance for those who responded to treatment. Patients with no detectable ctDNA after two cycles of induction exhibited significantly longer progression-free survival and overall survival compared to those with detectable ctDNA. This indicates that monitoring ctDNA post-induction can preemptively identify molecular relapses, often before clinical relapses, thus highlighting ctDNA’s potential as both a predictive and prognostic biomarker in MCL. The dynamic nature of ctDNA and its predictive capacity can be harnessed by continuously reassessing tumor burden during and following therapy, which may offer a more precise method for individualizing treatment compared to baseline factors [68].

Autologous stem cell transplantation (ASCT) has emerged as a promising treatment for MCL. The ability to predict a relapse post-autologous stem cell transplantation can enhance survival by enabling more intensive pre-transplant conditioning and post-transplant interventions. To this end, a study conducted by Wang et al. employed the sequencing of variable-diversity-joining (VDJ) recombination to detect MCL’s minimal residual disease in autologous grafts. The results indicate a direct correlation between higher loads of minimal residual disease and inferior post-ASCT progression-free survival and overall survival rates [69].

In a notable clinical trial, researchers performed comprehensive whole-exome sequencing analysis on MCL patients. The 5-year overall survival rates were stratified based on risk clusters, defined as distinctive genetic features with clinical implications. One cluster, characterized by mutated *IGHV*, *CCND1* mutation, amp(11q13), and active BCR signaling showed a 100% 5-year overall survival rate, indicating a more favorable prognosis. In contrast, Cluster C2, characterized by del(11q)/*ATM* mutations and the upregulation of NF-κB and DNA repair pathways, reflected genetic alterations associated with a distinct subset of MCL, resulting in a 5-year survival of 56.7%. Cluster C3, with mutations in *SP140*, *NOTCH1*, and *NSD2*, and the downregulation of BCR signaling and MYC targets, had a 5-year overall survival rate of 48.7%. Finally, Cluster C4, characterized by del(17p)/*TP53* mutations, del(13q), and del(9p), along with an active MYC pathway and hyperproliferation signatures, faced a more challenging prognosis, with a 5-year overall survival rate of 14.2%. This study elucidated the temporal order of genetic events and the clonal evolution of MCL, providing valuable insights into the impact of genetic changes on clinical outcomes [70].

The clinical implications of NGS in MCL are profound. The ability to precisely assess a patient’s risk profile using NGS data enables oncologists to tailor treatment strategies. Patients with high-risk genetic features may benefit from more aggressive therapeutic approaches, while those with low-risk features may be spared unnecessary interventions. Furthermore, the use of ctDNA for monitoring minimal residual disease allows for early intervention if disease progression is detected, ideally leading to improved clinical outcomes.

## 5. Novel Treatment Approaches Addressing the Biologic Heterogeneity of MCL

Advancements in MCL prognostication and genomic screening have helped to reveal the aforementioned pathophysiologic drivers that have served as therapeutic targets for recent pharmacotherapeutic innovations, such as the covalent BTK inhibitors (e.g., ibrutinib, acalabrutinib, and zanubrutinib), which have emerged as second-line modalities. Despite the availability of newer therapeutic agents and the potential for observation in a subset of patients with indolent disease, the mainstay of treatment for MCL is chemoimmunotherapy. Therapeutic decisions usually depend on the patient’s eligibility for transplant. Transplant-eligible patients receive induction chemotherapy with cytarabine and rituximab followed by autologous stem cell transplantation and rituximab maintenance. For patients ineligible for transplants, the mainstay of treatment is chemoimmunotherapy with bendamustine/rituximab [20]. The BTK inhibitors, as well as other recent modalities for relapsed/refractory (R/R) MCL, including BTK degraders, BCL-2 inhibitors, doublet and triplet regimens, ROR1 inhibitors, T-cell engagers, immunomodulators, and CAR-T therapies, are reviewed herein.

### 5.1. Covalent BTK Inhibitors

The advent of targeted therapies, notably BTK inhibitors, has prompted investigations into their potential to enhance or replace existing treatments. B-cell receptor signaling is essential for B-cell activation, survival, and antibody production. BTK plays a key role in this pathway. When dysregulated, this signaling pathway can lead to B-cell malignancies, such as MCL. The novel covalent BTK inhibitors are a class of medications which irreversibly bind to BTK, blocking its activity and preventing abnormal B-cell growth.

### 5.2. Ibrutinib

Table 4 summarizes key details from the TRIANGLE, SHINE, ENRICH, WINDOW-1, and WINDOW-2 trials, which explored the use of ibrutinib across various MCL settings [71,72,73,74,75]. While the outcomes of these trials were mostly positive, in April 2023, AbbVie, the United States manufacturer of ibrutinib, voluntarily withdrew the accelerated approval for its use in MCL and marginal zone lymphoma (MZL). This decision was made after the confirmatory phase III SHINE trial did not show the expected OS benefits [76]. Additionally, a parallel confirmatory trial in MZL, SELENE (NCT01974440), also failed to demonstrate the necessary survival benefits [77]. Although its use for MCL has been retracted, ibrutinib remains an important therapy for several B-cell malignancies, and has helped to promote the development of next-generation BTK inhibitors in MCL, hopefully with better efficacy and tolerability.

### 5.3. Acalabrutinib

Acalabrutinib is another oral covalent BTK inhibitor approved for the treatment of relapsed MCL, with a reduced incidence of off-target effects when compared to ibrutinib due to its higher selectivity. Acalabrutinib exerts its pharmacologic action by irreversibly inhibiting BTK by binding covalently to Cys481. Table 4 reviews information from the phase 2 ACE-LY-004 and phase 3 ECHO trials [78,79]. Acalabrutinib has shown efficacy in both relapsed and untreated MCL, improving PFS when combined with chemoimmunotherapy. While the OS remains inconclusive, its favorable response rates and manageable safety profile supports its role as a valuable treatment in MCL.

### 5.4. Zanubrutinib

Zanubrutinib is the most recent (2019) selective covalent BTK inhibitor to gain FDA approval for mantle cell lymphoma as a monotherapy [81]. Similar to the pharmacology of acalabrutinib, zanubrutinib was designed to minimize the inhibition of off-target kinases, while maintaining maximum exposure. Table 4 summarizes the durable efficacy and favorable risk–benefit profile associated with zanubrutinib in patients with R/R MCL, and further evaluation is merited to understand its utility in combination regimens [80].

### 5.5. Noncovalent BTK Inhibitor: Pirtobrutinib

Pirtobrutinib, a noncovalent BTK inhibitor, was studied in the multicenter phase I/II BRUIN trial which evaluated its efficacy in patients with MCL that had been pretreated with covalent BTK inhibitors (ibrutinib, acalabrutinib, zanubrutinib). Patients had received a median of three prior lines of therapy, 82.2% of whom had discontinued covalent BTK inhibitor treatment due to disease progression. The ORR in this population was 57.8%, with 20.0% of patients achieving CR. The median duration of response (DOR) was 21.6 months, with a 6-month estimated DOR of 73.6% and a 12-month estimated DOR of 57.1% [80]. It is theorized that noncovalent BTK inhibitors are designed to bind reversibly to both wild-type and mutant forms of the BTK receptor, maintaining efficacy even when covalent BTK inhibitors fail due to mutations at the binding site. As a result, pirtobrutinib, the first-in-class noncovalent BTK inhibitor approved by the FDA in 2023, is an effective therapeutic option for heavily pretreated R/R MCL and a notable treatment advancement given its capacity to demonstrate efficacy following covalent BTK inhibitor use [82,83].

### 5.6. BTK Degraders: BGB-16673 and NX-2127

BTK degraders represent a new approach to targeting BTK in B-cell malignancies. Unlike traditional BTK inhibitors, which covalently or noncovalently bind to BTK, BTK degraders function as a chimeric degradation activation compound that binds to BTK and recruits an E3 ubiquitin lipase. This leads to the ubiquitination and subsequent proteasome degradation of BTK, leading to the degradation of both wild-type and mutated BTK. By degrading rather than inhibiting its activity, this class offers the potential to overcome the resistance mechanisms that plague current BTK inhibitors. Investigational BTK inhibitors include BGB-16673, which has received a Fast Track designation for CLL/SLL, and NX-2127, a bifunctional molecule that promotes both BTK degradation and immunomodulation [84,85]. Early clinical data show promising efficacy and safety for BGB-16673 in R/R B-cell malignancies including MCL, while the preclinical assessment of NX-2127 shows ~80% reductions in BTK levels in peripheral blood [85,86,87]. Further clinical evaluations of BTK degraders across MCL and other B-cell malignancies are ongoing.

### 5.7. BCL-2 Inhibitors

#### 5.7.1. Venetoclax

B-Cell Lymphoma-2 (BCL-2), a pro-oncogene, is an important regulator of apoptosis in normal cells, acting at the mitochondrial membrane to block the intrinsic pathway. In healthy cells, it binds and sequesters pro-apoptotic proteins, thereby preventing apoptosis. In MCL—as well as other B-cell malignancies—the over-expression of BCL-2 disrupts the balance between survival and cell death. The aberrant expression of BCL-2 enables malignant cells to evade apoptosis, but also contributes to resistance against conventional chemotherapies. The development of selective BCL-2 inhibitors has become an attractive target for novel therapies, which aim to restore the natural apoptotic pathway.

Venetoclax, a selective BCL-2 inhibitor, was initially studied in CLL, demonstrating impressive efficacy and gaining approval in 2016. Its success led to further research in MCL, where early trials showed promising results. Early phase studies of venetoclax monotherapy in R/R MCL reported encouraging outcomes—a phase I study reported an ORR of 75% and CR of 21% [88]. Another study found that R/R MCL treated with venetoclax monotherapy had an ORR of 53% and CR of 18% [89].

Combining BTK inhibitors with BCL-2 inhibitors was the logical next step to synergistically inhibit two different pathways that promote uncontrolled cancer growth. The phase II AIM study (NCT02471391) evaluated this combination of venetoclax and ibrutinib in high-risk MCL patients. At an early time point (week 16), the study reported a high CR at 62% and OR at 71% [90]. Importantly, this same study with long-term follow up demonstrated durable outcomes, including an impressive 7-year PFS of 30%, OS of 43%, and a median DOR of 81 months [91]. In parallel, the phase III SYMPATICO trial (NCT03112174) compared the ibrutinib–venetoclax combination regimen against ibrutinib alone in R/R MCL patients with *TP53* mutations. It reported a similar PFS to the AIM study, lending further credence to the concept that combining BCL-2 inhibition with BTK inhibition improves clinical outcomes over single-agent approaches [92,93].

Building on the promising results of AIM and SYMPATICO, the AIM 2 trial (NCT05864742) and TRAVERSE (NCT05951959) are currently refining the combination treatment strategies through genomic risk stratification. The AIM 2 trial tailors treatment based on a genetically risk-stratified approach: those without high-risk markers receive a combination of ibrutinib, rituximab, and venetoclax; those with high-risk features additionally receive navitoclax [92]. Conversely, the TRAVERSE trial is evaluating a fixed-duration target agent combination of acalabrutinib, venetoclax, and rituximab. Patients who achieve a CR with undetectable minimal residual disease (MRD) are randomized to either continue maintenance therapy or undergo observation [94]. Collectively, the AIM, AIM2, SYMPATICO, and TRAVERSE trials have and will continue to contribute toward our understanding of how targeted combinations can improve outcomes in R/R MCL.

#### 5.7.2. Sonrotoclax

Sonrotoclax, a second-generation BCL-2 inhibitor, was developed to address the limitations seen in venetoclax. Sonrotoclax has been shown to have a higher potency and selectivity than venetoclax. Additionally, sonrotoclax also addresses the emerging resistance mechanisms associated with earlier BCL-2 inhibitors. Early data show that sonrotoclax can overcome the G101V-mediated resistance observed with venetoclax [95].

Recent clinical investigations have highlighted the promise of sonrotoclax in the treatment of R/R MCL. A phase I study evaluated combination therapy with sonrotoclax and zanubrutinib. Patients achieved an impressive ORR of 92% (83% CR) after 12.5 months. Notably, the combination therapy was well tolerated. Adverse effects such as neutropenia were observed but managed effectively, and no dose-limiting toxicities, atrial fibrillation, or tumor lysis syndrome events were reported [96]. Future pivotal studies will be key to establishing its definitive place in MCL treatment protocols.

### 5.8. ROR1 Inhibitors

#### Zilovertamab

Receptor tyrosine kinase-like orphan receptor 1 (ROR1) is a tyrosine kinase implicated in the regulation of cell growth and survival. ROR1 functions as a receptor for the noncanonical ligand Wnt5a, triggering signaling pathways that activate NF-κB, Cac1, and other downstream pathways, resulting in cell survival, proliferation, and resistance to apoptosis. High levels of ROR1 were first discovered in CLL, but are now being identified in other hematologic malignancies including MCL [97]. Given its expression in malignant cells, ROR1 represents an attractive target for therapeutic intervention.

Zilovertamab (also marketed as cirmtuzumab), a humanized monoclonal antibody that inhibits ROR1, has previously undergone evaluation for the treatment of MCL. A phase I/II clinical trial explored the combination of cirmtuzumab with ibrutinib in patients with B-cell malignancies, including R/R MCL. Among the MCL patients, cirmtuzumab-based therapy had an ORR of 82%, with 41% achieving CR. These responses occurred rapidly, typically within 3 months of initiating treatment, and were durable, notably lasting 8 to 28+ months [98].

A phase I trial investigated the efficacy of zilovertamab vedotin, an antibody–drug conjugate in patients with R/R lymphoid cancers, including MCL. It contains the anti-microbule cytotoxin monomethyl auristatin E, a potent cytotoxic agent. The small study reported a 47% objective tumor response in patients with MCL. The safety profile was consistent with what is seen with cytotoxic agents: neuropathy and neutropenia.

Unfortunately, due to finances, the company behind its development, Oncternal Therapeutics, has paused two clinical trials (NCT03088878 and NCT05431179), halting further investigation of zilovertamab vedotin in MCL [99]. While zilovertamab vedotin has potential, its future is unclear.

### 5.9. T-Cell Engagers

#### 5.9.1. Blinatumomab

The use of T-cells to enhance antitumor activity has emerged as a strategy in the treatment of hematological malignancies. T-cell engager (TCE) antibodies are one such modality that has shown efficacy in the treatment of different hematological malignancies [100]. TCEs exert their pharmacologic action by binding endogenous T-cells to tumor-expressed antigens, leading to the activation of the cytotoxic potential of T-cells to enable tumor cell killing. One binding site is specific for a tumor-associated antigen, while the other binds to CD3 receptors on T-cells. By simultaneously engaging both the T-cell and cancer cell, it effectively creates an artificial, immunological synapse triggering T-cell activation. However, because TCEs lack an Fc region, they do not possess some of the effector functions seen in other conventional antibody therapies, and have a shorter half-life.

Blinatumomab (CD19-BiTE), the first approved TCE molecule, targets and binds CD19 surface antigens on B-cells. It is currently used in the treatment of R/R B-cell precursor ALL and has shown promising response rates in a subset of MCL patients who received blinatumomab in a phase I study [101]. Another study provided insights into the durability of response with blinatumomab, with an ORR of 46.2% in the MCL subgroup. The most frequent adverse effects were consistent with T-cell activation, including flu-like symptoms and fatigue [102].

#### 5.9.2. Epcoritamab

Epcoritamab is a novel CD3xCD20 T-cell-engaging bispecific antibody that induces T-cells to kill malignant CD20-positive B-cells. Unlike traditional TCEs, epcoritamab contains an Fc region which extends the half-life of the medication. Originally investigated in R/R DLBCL, epcoritamab showed an ORR of 63.1% and a CRR of 38.9% [103].

Limited data are available for epcoritamab in the treatment of MCL. In a study of epcoritamab for the treatment of R/R NHL (i.e., DLBCL, FL, MCL), a sub-analysis of 4 patients with MCL showed that epcoritamab was associated with an ORR of 50%, including a 25% CRR [104]. These results support further investigation in MCL-specific trials to define its efficacy and durability.

#### 5.9.3. Glofitamab

Glofitamab is a CD3xCD20 T-cell-engaging bispecific antibody. However, unlike epcoritamab, it is engineered in a 2:1 format, possessing two binding sites for CD20 and one for CD3, enhancing its tumor cell avidity. It is also a full-length IgG and offers an extended half-life. In a phase I/II study (NCT03075696), it has achieved an impressive ORR and CR in patients who failed prior lines of therapy. Specifically, patients with R/R MCL, including patients with previous treatment with a BTK inhibitor, were pretreated with the humanized anti-CD20 monoclonal antibody obinutuzumab to minimize cytokine release syndrome. Afterwards, patients were treated with glofitamab. The CR rate was 78.3% while the ORR was 85.0%. Patients who received BTK inhibitors in the past had a CR of 71% and ORR of 74.4%. The PFS of all patients was 16.8 months. While 70% of patients experienced CRS, 58.3% of events were grade 1/2 [105,106]. Currently, a phase III trial, GLOBRYTE (NCT06084936), is evaluating glofitamab monotherapy compared to rituximab with bendamustine or lenalidomide [107].

#### 5.9.4. Mosunetuzumab

Mosunetuzumab, a CD3xCD20 T-cell-engaging bispecific antibody, has demonstrated promising efficacy (ORR 44%; CR 24%) in a phase I/II trial that evaluated it as a monotherapy in heavily pretreated R/R mantle cell lymphoma (MCL) patients who previously received treatment with BTK inhibitors. CRS occurred in half of patients, with most cases being Grade 1/2 [108]. Mosunetuzumab has also been evaluated with polatuzumab vedotin in patients with R/R MCL who had received two or more prior lines of therapy including a BTK inhibitor, with results showing an ORR of 75% with a 70% CR and a consistent efficacy in high-risk subgroups, with a safety profile characterized by low-grade adverse events [109].

#### 5.9.5. NVG-11

NVG-11, a novel, humanized, ROR1-CD3 bispecific T-cell engager, is undergoing evaluation in a phase I, first-in-human, dose-escalation study in patients with CLL and MCL. Those who previously responded to ibrutinib received NVG-11/ibrutinib while those who progressed on BTK inhibitor- or BCL-2 inhibitor-based therapy received NVG-11 monotherapy. The results showed increased cytotoxic CD8+ T-cell activation marker expression without exhaustion, with one MCL patient achieving CR. Additional trials are being undertaken to further evaluate the therapeutic promise of NVG-11 [110].

### 5.10. Immunomodulatory Agents: Lenalidomide

Immunomodulatory agents (IMiDs) have become an integral part of the treatment landscape for several hematologic malignancies, including MCL. The first IMiD, thalidomide, was discovered in the early 1950s, and was introduced as a sedative and antiemetic, widely prescribed to pregnant women to treat morning sickness. Unfortunately, it became notorious due to its association with severe birth defects and was thus withdrawn from the market. It re-emerged in the 1990s where it was found to have significant immunomodulatory and anti-inflammatory properties. Researchers were able to modify thalidomide’s structure to enhance its anti-cancer properties while reducing its side effects, leading to the creation of lenalidomide, a second-generation IMiD.

Lenalidomide exerts its anti-neoplastic effects through a combination of direct cytotoxic, immunomodulatory, and anti-angiogenic activity. It induces T-cell and NK-cell activation, leading to an improved immune-mediated attack on tumor cells, the inhibition of tumor growth factors inducing apoptosis, and the inhibition of angiogenesis [111].

The EMERGE study (MCL-001) evaluated lenalidomide as monotherapy in patients with MCL who had been previously treated with rituximab, cyclophosphamide, anthracycline/mitoxantrone, and bortezomib alone or in combination. This study reported an ORR of 28% with a CR of 7.5%, and a median PFS of 4 months. This led to the approval of lenalidomide for relapsed/refractory MCL [112]. Following the success of the EMERGE study, the SPRINT study (MCL-002) compared lenalidomide to the investigator’s choice of single-agent therapy in patients with R/R MCL. The trial demonstrated the superiority of lenalidomide, with a median PFS of 8.7 months versus 5.2 months for the investigator’s choice [113]. In addition to monotherapy, the combination of lenalidomide with rituximab was studied, with investigators identifying 3-year PFS and an OS of 80% and 90%, respectively [114].

Lenalidomide has also been studied in the maintenance therapy setting following induction chemotherapy or autologous stem cell transplant. A phase 3 trial investigated lenalidomide maintenance for 24 months, or observation. The 3-year PFS was significantly higher in the lenalidomide group at 80% compared to 64% in the observation group, indicating a 49% risk reduction of progression or death [115].

### 5.11. Doublet and Triplet Regimens: Lenalidomide–Rituximab and Acalabrutinib–Lenalidomide–Rituximab

Although the standard of care for MCL has traditionally been chemotherapy/immunotherapy, recent studies have explored chemotherapy-free approaches. A phase II study (NCT01472562) investigated lenalidomide and rituximab as the initial treatment for MCL. Disease progression occurred in fifteen patients (three during the induction phase, and twelve during the maintenance phase), and after a median follow up of 106 months, seventeen patients remained in remission. It showed durable outcomes, with a 9-year PFS and OS of 51% and 66%, respectively [114,116].

Following the promising results of lenalidomide plus rituximab in treating MCL, a subsequent phase II study (NCT03863184) investigated the addition of a second-generation BTK inhibitor—acalabrutinib—to either lenalidomide plus rituximab or obinutuzumab for patients with treatment-naive stage III/IV MCL. After a median follow-up of 41 months, the rituximab group had a 3-year OS and PFS of 95.2% and 87.5%, respectively. These results suggest the promising efficacy of doublet and triplet regimens as an initial treatment for MCL [117].

### 5.12. CAR-T Therapy

Chimeric antigen receptor T-cell (CAR-T) therapy represents a significant advancement in the treatment of B-cell malignancies, including MCL. CAR-Ts are T-cells that are genetically engineered to express synthetic receptors, known as chimeric antigen receptors (CARs), which specifically target tumor antigens. This modification enables the T-cells to recognize and destroy cancerous cells with precision, accuracy, and potency. The most common target for CAR-T therapies is the CD19 antigen, which is widely expressed on surface B-cells, including those in MCL.

The CAR construct consists of an extracellular antigen-binding domain, a transmembrane domain, and one or more intracellular signaling domains that activate the T-cell upon antigen recognition. A critical component of CAR design is the inclusion of costimulatory domains within the intracellular signaling region. These costimulatory domains are essential for enhancing the efficacy of CAR-Ts. The most commonly used costimulatory domains are CD28 and 4–1BB (CD137), each influencing the function of CAR-Ts in distinct ways.

Table 5 summarizes additional important CAR-Ts in relation to MCL. The approval of Brexucabtagene Autoleucel (KTE-X19, Tecartus^®^) for MCL marked a significant advancement in the treatment of this aggressive malignancy. The ZUMA-2 trial evaluated the efficacy and safety of Brexucabtagene Autoleucel in patients with R/R MCL who have received at least two prior therapies, including a BTK inhibitor. The trial demonstrated an impressive ORR of 87%, with a CRR of 62%. These results led to the FDA approval of Brexucabtagene Autoleucel for this indication [118].

The TRANSCEND trial investigated Lisocabtagene Maraleucel (Breyanzi^®^) across various B-cell lymphomas. Although this trial primarily studied DLBCL, it included a subset of patients with MCL, where the therapy showed an ORR of 83% and a CR rate of 72.3% in the MCL cohort [119].

Despite the promising results of CAR-T therapies, patients still experience relapses. Understanding the mechanisms underlying resistance and relapse is crucial for improving outcomes. Variability in the manufacturing process can affect the potency and durability of the CAR-Ts. The conditioning chemotherapy regimen influences the environment for CAR-Ts, and antigen escape through the deregulation or loss of CD19 expression renders CD19-targeted CAR-Ts ineffective. The increased expression of T-cell exhaustion markers, including PD-1, TIM-3, LAG-3, and others, leads to decreased proliferation, decreased cytokine production, and decreased activity of the T-cells [120].

**Table 5:** CAR-T therapies for mantle cell lymphoma.

Trial Name	Therapy	Line of Therapy	Phase	Total Patients	MCL Patients	Key Findings	Status
ZUMA-2 [118]	Brexucabtagene autoleucel (KTE-X19, Tecartus^®^)	≥3rd	Phase 2	74	74	Achieved an ORR of 87%, with a CR rate of 62%.	Completed
TRANSCEND [119,121]	Lisocabtagene maraleucel (Breyanzi^®^)	≥3rd	Phase 1	344	88	Achieved an ORR of 83% and a CR rate of 72.3% in the MCL cohort.	Completed
TARMAC study [122]	Tisagenlecleucel (CTL019, Kymriah^®^) + Ibrutinib	≥2nd	Phase 2	20	20	12-month PFS and overall survival were 75% and 100%.	Completed
NCT04484012 [123]	CD19.28.z.EGFRt-CAR + acalabrutinib	≥3rd	Phase 1	8	8	Achieved an ORR of 88%, with a CR rate of 75%.	Completed

CAR = chimeric antigen receptor; CAR-T = chimeric antigen receptor T-cell; CD19 = cluster of differentiation 19; CR = complete response; EGFRt = truncated human epidermal growth factor receptor; MCL = mantle cell lymphoma; ORR = objective response rate; PFS = progression-free survival.

### 5.13. Rituximab Maintenance in MCL

Maintenance rituximab (MR) in MCL is used with the goal to prolong remission, though its benefit appears to vary by treatment setting and patient population. The MAINTAIN trial (NCT00877214) evaluated the impact of MR compared to observation following first-line BR with previously untreated MCL. After a median follow-up of 4.5 years, there were no significant differences in PFS or OS between the MR and observation groups [124]. However, a retrospective study indicated an impressive benefit, with a longer time to the next treatment and an improved overall survival in patients receiving MR. Specifically, the 3-year PFS and OS in the MR group were 74.2% and 91.9%, respectively, compared to 48.5% and 73.2% in those without maintenance therapy [125].

Another observational study analyzed the impact of MR in patients who received BR, demonstrating that those on maintenance therapy experienced significantly prolonged event-free survival and OS compared to observation alone. Notably, the subgroup analysis suggested that the benefit for MR was most pronounced in patients who achieved CR following induction therapy, reinforcing the idea that deeper initial responses may predict greater benefits from maintenance treatment [126].

For younger, newly diagnosed MCL patients who responded to R-DHAP induction and underwent autologous stem cell transplant (ASCT), the phase III LYMA trial (NCT00921414) provided strong evidence for the role of MR after ASCT. This study showed that MR significantly prolonged PFS, with 7-year estimates of 78.5% compared to 47.4% in the observation group. While the 7-year OS estimate favored MR (83.2% vs. 72.2%), the difference was not statistically significant [127].

These findings suggest that the efficacy of MR may be closely linked to the depth of the initial response, with patients achieving MRD negativity potentially deriving the most benefit. NGS provides critical insights into MRD status, which could help clinicians to personalize maintenance strategies to maximize the likelihood of long-term remission. Incorporating NGS into future studies investigating the role of maintenance regimens, including MR, in MCL will enable investigators to assess this approach in a manner inclusive of the latest technology.

## 6. Navigating the Tumor Microenvironment in MCL

Research into the tumor microenvironment (TME) in MCL has lagged behind that of other B-cell malignancies. Nonetheless, recent studies have established a link between malignant B-cells and TME components within lymph nodes. This interaction modulates critical cellular survival and proliferation pathways, including BCR-mediated signaling and NF-kB pathways [128]. Therefore, the efficacy of BTK inhibitors, which exert their pharmacologic action on the BCR signaling pathway, may be impacted by TME. A comprehensive profile analysis shed light on this interaction by identifying an immune depleted MCL subtype. This subtype is characterized by the presence of high-risk somatic mutations that confer inferior OS, the reduced expression of major histocompatibility complex I and II transcripts, both primary and acquired resistance to BTK inhibitors, and the overexpression of PI3K [128,129].

Emerging evidence supports that combining targeted agents may overcome TME-mediated drug resistance. For instance, studies have shown that BCL-2 inhibitor resistance may be related to the upregulation of the anti-apoptotic protein BCL-XL [130]. This effect can be countered by the simultaneous use of BCL-2 inhibitors and BTK inhibitors. This mechanism is supported by clinical trials, including the AIM trial, which combines ibrutinib and venetoclax in R/R MCL and has shown impressive response rates with manageable toxicity profiles [93].

Further emphasizing the TME’s role, immune profiling has revealed that higher CD4:CD8 ratios have been found to be associated with indolent disease. Flow cytometric analysis has identified high CD4:CD8 ratios as an independent predictor of a longer OS [131]. A deeper understanding of the TME in MCL is crucial, not only for prognostication but also for designing more effective treatment regimens.

## 7. Conclusions and Recommended Treatment Approaches

MCL is a complex and heterogeneous disease, characterized by a broad spectrum of clinical behaviors ranging from indolent to highly aggressive forms. Over the past decade, significant advancements in genomic profiling and prognostication have deepened our understanding of the biologic underpinnings of MCL, leading to the development of targeted therapies that address the unique pathophysiological drivers of the disease. The application of NGS is revolutionizing the landscape of MCL management, allowing for more precise risk stratification and personalized treatment strategies. Through the identification of key genetic mutations and the utilization of ctDNA as a biomarker, oncologists can now more accurately predict patient outcomes and tailor treatment regimens accordingly.

Among the novel therapeutic approaches, BTK inhibitors, such as acalabrutinib and zanubrutinib, have emerged as critical components in the treatment of relapsed/refractory MCL. These agents have demonstrated robust efficacy, particularly in high-risk patients, and have shown potential when combined with other therapies to improve outcomes while minimizing chemotherapy-related toxicity. Additionally, newer modalities like pirtobrutinib, a noncovalent BTK inhibitor; BTK degraders; and zilovertamab (cirmtuzumab), a ROR1-targeting monoclonal antibody, offer promising options for heavily pretreated patients, particularly those who have progressed on standard therapies. The exploration of T-cell engagers, such as blinatumomab, epcoritamab, glofitamab, mosunetuzumab, and NVG-11, as well as CAR-T modalities, further exemplifies the potential of immunotherapy in MCL, providing innovative approaches to leverage the immune system in targeting malignant cells. These interventions, as well as other modalities, including lenalidomide, maintenance rituximab, and doublet and triplet regimens, continue to be explored.

Despite these advancements, chemoimmunotherapy remains the cornerstone of MCL treatment, particularly for transplant-eligible patients. For transplant-ineligible patients, bendamustine–rituximab remains a standard of care, with emerging data supporting the integration of BTK inhibitors to enhance efficacy and reduce treatment-related toxicity.

Looking forward, ongoing research into the molecular drivers of MCL and the continued development of targeted therapies hold the promise of further improving patient outcomes. The integration of genomic data into clinical decision-making, combined with the judicious use of novel agents, is likely to refine the therapeutic landscape of MCL, offering hope for more durable remissions and a better quality of life for patients with this challenging disease.

## Figures and Tables

**Table 1:** Updated classification of mantle cell lymphoma (MCL).

Subtype	Clinical Presentation	Molecular Genetics	Ki-67%	Clinical Course
In situ mantle cell neoplasia	Asymptomatic and/or without B symptoms	Cyclin D1 (+)	Low	Indolent, low risk of progression
Classic MCL	Worsening lymphadenopathy, B symptoms	SOX11 (+) Cyclin D1 (+) Unmutated IGHV	High	Aggressive
Non-nodal MCL	Lymphocytosis, splenomegaly, extranodal symptoms, rare lymphadenopathy	SOX11 (−) Mutated IGHV	Low	Indolent

**Table 2:** Variants of classical MCL.

Subtype	Key Genetic Abnormality	Cyclin D1 Status	Notable Features
Classical MCL	t(11;14)(q13;q32)/*IGH:CCND1* Translocation	Positive	Usually arises from mature B-cells Limited germinal center involvement Typically unmutated *IGHV* SOX11 overexpression Mutations in *ATM*, *CDKN2A*, various chromatin modifiers [16,17]
Cyclin D1-Negative	Various rearrangements (i.e., *CCND2*, *CCND3*)	Negative	Alternate mechanism for cell cycle dysregulation Less common but important to distinguish [18]
Blastoid/Polymorphic	Complex karyotype with chromosomal instability *TP53*, *NOTCH1*, *NOTCH2*	Typically Positive	Aggressive clinical behavior High Ki-67 index Morphological resemblance to lymphoblastic lymphoma Poor prognosis [19,20,21]

*ATM =* ataxia-telangiectasia mutated; *IGHV* = immunoglobulin heavy chain variable; *CCND1* = cyclin D1; *CCND2* = cyclin D2; *CCND3* = cyclin D3; *CDKN2A* = cyclin-dependent kinase inhibitor 2A; *TP53* = tumor protein 53.

**Table 3:** Clinical standard prognostic risk scoring systems in mantle cell lymphoma.

Scoring System	IPI [26]	MIPI [24]	s-MIPI [27]	MIPI-b [24]	MIPI-B-miR [28]	MIPI-c [29]
Components	Age, LDH, Performance Status, Ann Arbor Stage, Extra-nodal Involvement	Age, ECOG, Serum LDH, WBC Count	Age, ECOG, LDH, WBC Count	Age, ECOG, LDH, WBC Count, Ki67%	MIPI-b, miR-18b	Age, ECOG, LDH, WBC Count, Ki-67%
Points and Calculation	One point for each of the following risk factors: Age > 60 Serum LDH level > ULN ECOG performance> 2 Ann Arbor stage III–IV >1 extranodal site	MIPI = (0.03535 × age) + 0.6978 (if ECOG > 1) + [1.367 × log10 (LDH/ULN) + 0.9393 × log10 (WBC)]	0 points: Age < 50, ECOG 0–1, LDH < 0.67 ULN, WBC < 6.7 1 point: Age 50–59, LDH 0.67–0.99 ULN, WBC 6.7–9.99 2 points: Age 60–69, ECOG 2–4, LDH 1.0–1.49 ULN, WBC 10–14.99 3 points: Age ≥ 70, LDH > 1.5 ULN, WBC ≥ 15	(0.03535 × age) + 0.6978 (if ECOG > 1) + [1.367 × log10 (LDH/ULN) + 0.9393 × log10 (WBC)] + 0.0214 × Ki67%	MIPI-B + 0.58317 × log_2_(fold change in miR-18b)	MIPI + Ki67% (<30% or ≥30%)
Risk Groups	0–1: Low 2: Low-Intermediate 3: High-Intermediate 4–5: High	Low Risk: <5.7 Intermediate Risk: ≥5.7 High Risk: ≥6.2	0–3: Low 4–5: Intermediate 6–11: High	<5.7: Low ≥5.7 and <6.5: Intermediate ≥6.5: High	<5.75: Low ≥5.75 and ≤7.49: Intermediate >7.49: High	Low: LR MIPI + Ki67 <30% Low-Intermediate: either LR MIPI + KI67 ≥ 30% or IR MIPI + KI67 < 30% High Intermediate: either IR MIPI + Ki67 ≥ 30% or HR MIPI + KI67 < 30% High: HR MIPI + Ki67 ≥ 30%
Relevance	Poorly differentiates the high-intermediate and high-risk categories. Less specific for MCL but still useful in broader lymphoma prognostication. [25]	Widely accepted and validated in multiple studies, making it the standard tool in both clinical practice and research.	Useful for quick risk assessment without needing full lab workups. Similar prognostic performance to that of MIPI. [24]	Enhances MIPI by integrating genetic factors. However, consistently reports a small low-risk group which has overlap with the intermediate risk-group, effectively making it a two-group stratification system. [29]	Integrates molecular profiling into the MIPI-B, enhancing the prognostic information. Useful for personalized treatment approaches. [28]	More precise risk stratification than the MIPI alone, particularly useful in identifying high-risk patients. [29]
Survival	Over 50% 5-year survival in the high-intermediate and high-risk categories [25]	35% 5-year survival in the high-risk group [25]	38% 5-year survival in the high-risk group [25]	46% 5-year survival in the high-risk group [29]	20% 5-year survival in the high-risk group [28]	31% 5-year survival in the high-risk group [5]

ECOG = Eastern Cooperative Oncology Group; HR = high risk; IPI = International Prognostic Index; IR = intermediate risk; LDH = lactate dehydrogenase; LR = low risk; MCL = mantle cell lymphoma; MIPI = Mantle Cell Lymphoma International Prognostic Index; MIPI-B = Biological Mantle Cell Lymphoma International Prognostic Index; MIPI-B-miR = Biological Mantle Cell Lymphoma International Prognostic Index including mIR-18b expression; MIPI-c = Combined Mantle Cell Lymphoma International Prognostic Index; s-MIPI = Simplified Mantle Cell Lymphoma International Prognostic Index; ULN = upper limit of normal; WBC = white blood cell.

**Table 4:** Summarizing key findings from trials of BTK inhibitors.

Trial Name	Therapy	Phase	Patients	Key Efficacy Findings	Key Safety Findings
Ibrutinib					
TRIANGLE [71]	Standard therapy (i.e., chemotherapy ^a^ followed by ASCT) vs. ibrutinib + standard therapy vs. ibrutinib + chemotherapy alone	3	Advanced (stage II–IV) MCL in patients aged 65 or younger	3-year FFS: Standard therapy: 72% Ibrutinib + standard therapy: 88% Ibrutinib + chemotherapy alone: 86%	Similar risk of grade 3–5 events during initial treatment Ibrutinib increased risk of infections, neutropenia, and cardiac issues during maintenance
SHINE [72,76,77]	Ibrutinib + BR (IBR) with MR vs. BR with MR	3	Previously untreated stage II–IV MCL in patients aged 65 or older	Median PFS: IBR with MR: 80.6 months BR with MR: 52.9 months Overall survival: No statistical significance between groups	Increased risk of hematologic toxicities, atrial fibrillation, and infections with ibrutinib addition AbbVie voluntarily recalled ibrutinib in April 2023 due to shortcomings in overall survival
ENRICH [73]	Ibrutinib + rituximab (IR) vs. chemoimmunotherapy (R-CHOP or BR)	2/3	Previously untreated MCL in patients aged 60 or older	PFS: IR: 65.3 months R-CHOP or BR: 42.4 months	Lower hematologic toxicity and better quality of life with IR
WINDOW-1 [74]	IR induction (12 cyc + R-HCVAD alternating with MTX-ARA-C consolidation	2	Previously untreated MCL in patients aged 65 or younger	ORR (after induction): 98% (87% CR); similar efficacy in high- vs. low-risk Ki-67 and in pleomorphic/blastoid vs. classical MCL ORR (after consolidation): 90% (90% CR); significantly lower CR after induction with *TP53* aberrations	Most adverse events were grade 1/2 Most common grade 3–4 hematological and non-hematological adverse events were lymphocytopenia and rash, infection, and fatigue
WINDOW-2 [75]	IR followed by IRV + short-course MTX-ARA-C consolidation ^b^ + IRV maintenance	2	Previously untreated MCL	ORR: 96% (92% CR) after consolidation; similar rates regardless of risk category	Grade 3–4 toxicities included myelosuppression, fatigue, myalgia, rash, mucrositis, atrial flutter 3 on-trial deaths (6%); 2 off-trial deaths (progressive disease; COVID-19 pneumonia)
Acalabrutinib					
ACE-LY-004 [78]	Acalabrutinib	2	R/R MCL with one or more prior lines of therapy	ORR: 81.5%; consistent efficacy among high-risk subgroups (blastoid/pleomorphic; high-risk MIPI; Ki-67 > 50%)	66.1% Grade 3 or higher events, with serious adverse events in 50% (pneumonia being most common); highest risk in first year
ECHO [79]	Acalabrutinib + BR vs. placebo + BR MR initiated if CR or PR	3	Previously untreated MCL in patients aged 65 or older with ECOG 0–2	ORR: 91% vs. 88% mPFS: 66.4 months vs. 49.6 months	Comparable incidence of Grade 3 or higher and serious events (88.9% vs. 88.2%; 64.3% vs. 55.9%)
Zanubrutinib					
NCT03206970 [80]	Zanubrutinib	2	MCL with one or more prior lines of therapy	ORR: 83.7% (77.9% CR)	Predominantly Grade 1–2 events (e.g., neutropenia, URTI, rash, leukopenia, thrombocytopenia), with frequent onset in first 6 months

^a^ Chemotherapy regimen consisted of R-CHOP/rituximab/dexamethasone/cytarabine/cisplatin (R-DHAP) (or rituximab/dexamethasone/cytarabine/oxaliplatin [R-DHAOx]). ^b^ Consolidation regimen stratified by risk category: high-risk patients received four cycles; intermediate-risk patients received two cycles; low-risk patients received 0 cycles. ASCT = autologous stem cell transplantation; BR = bendamustine/rituximab; BTK = Bruton’s tyrosine kinase; CR = complete response; ECOG = Eastern Cooperative Oncology Group; FFS = failure-free survival; IRV = ibrutinib/rituximab/venetoclax; MCL = mantle cell lymphoma; MIPI = Mantle Cell Lymphoma International Prognostic Index; mPFS = median progression-free survival; MR = maintenance rituximab; MTX-ARA-C = methotrexate/cytarabine; ORR = overall response rate; PFS = progression-free survival; PR = partial response; R-CHOP = rituximab/cyclophosphamide/doxorubicin/vincristine/prednisone; R-HCVAD = rituximab/hyper-fractionated cyclophosphamide/vincristine/doxorubicin/dexamethasone; R/R = relapsed/refractory; URTI = upper respiratory tract infection.
